# Development and Characterization of a Camelid Single Domain Antibody–Urease Conjugate That Targets Vascular Endothelial Growth Factor Receptor 2

**DOI:** 10.3389/fimmu.2017.00956

**Published:** 2017-08-21

**Authors:** Baomin Tian, Wah Yau Wong, Marni D. Uger, Pawel Wisniewski, Heman Chao

**Affiliations:** ^1^Helix BioPharma Corp., Toronto, ON, Canada; ^2^Helix ImmunoOncology, Warsaw, Poland

**Keywords:** antibody–drug conjugates, conjugation sites, conjugation ratios, camelid antibodies, urease, vascular endothelial growth factor receptor 2, cancer

## Abstract

Angiogenesis is the process of new blood vessel formation and is essential for a tumor to grow beyond a certain size. Tumors secrete the pro-angiogenic factor vascular endothelial growth factor, which acts upon local endothelial cells by binding to vascular endothelial growth factor receptors (VEGFRs). In this study, we describe the development and characterization of V21-DOS47, an immunoconjugate that targets VEGFR2. V21-DOS47 is composed of a camelid single domain anti-VEGFR2 antibody (V21) and the enzyme urease. The conjugate specifically binds to VEGFR2 and urease converts endogenous urea into ammonia, which is toxic to tumor cells. Previously, we developed a similar antibody–urease conjugate, L-DOS47, which is currently in clinical trials for non-small cell lung cancer. Although V21-DOS47 was designed from parameters learned from the generation of L-DOS47, additional optimization was required to produce V21-DOS47. In this study, we describe the expression and purification of two versions of the V21 antibody: V21H1 and V21H4. Each was conjugated to urease using a different chemical cross-linker. The conjugates were characterized by a panel of analytical techniques, including SDS-PAGE, size exclusion chromatography, Western blotting, and LC-MS^E^ peptide mapping. Binding characteristics were determined by ELISA and flow cytometry assays. To improve the stability of the conjugates at physiologic pH, the pIs of the V21 antibodies were adjusted by adding several amino acid residues to the C-terminus. For V21H4, a terminal cysteine was also added for use in the conjugation chemistry. The modified V21 antibodies were expressed in the *E. coli* BL21 (DE3) pT7 system. V21H1 was conjugated to urease using the heterobifunctional cross-linker succinimidyl-[(*N*-maleimidopropionamido)-diethyleneglycol] ester (SM(PEG)_2_), which targets lysine resides in the antibody. V21H4 was conjugated to urease using the homobifunctional cross-linker, 1,8-bis(maleimido)diethylene glycol (BM(PEG)_2_), which targets the cysteine added to the antibody C-terminus. V21H4-DOS47 was determined to be the superior conjugate as the antibody is easily produced and purified at high levels, and the conjugate can be efficiently generated and purified using methods easily transferrable for cGMP production. In addition, V21H4-DOS47 retains higher binding activity than V21H1-DOS47, as the native lysine residues are unmodified.

## Introduction

As tumors outgrow local oxygen diffusion gradients, angiogenesis is triggered and new capillaries sprout from pre-existing blood vessels to support tumor cell demands for nutrients and oxygen and spread to distant sites (1, 2). Growth factors secreted by tumor cells mediate the induction of angiogenesis and control the inflammatory infiltrate. Multiple ligand–receptor signaling networks have been associated with tumor angiogenesis. The vascular endothelial growth factor (VEGF)/vascular endothelial growth factor receptor 2 (VEGFR2) ligand–receptor complex is one of the most important signaling pathways identified and extensive research has been done on its roles in vascular functions (3, 4). During cancer-induced angiogenesis, cancer cells secrete VEGF that binds to VEGFR2, triggering a tyrosine kinase signaling cascade *via* the dimerization of VEGFR2. Consequently, the kinase signaling cascade stimulates the production of factors that variously stimulate vessel permeability, proliferation/survival, migration, and finally differentiation of mature blood vessels. Tumor blood vessels are structurally and functionally abnormal and are present at high density. Therefore, targeting tumor vasculature is a rational strategy with great promise and different anti-angiogenic agents targeting the VEGF/VEGFR2 signaling cascade have been developed as cancer therapeutics. Small chemical drugs, such as Sunitinib (SU11248, Sutent, Pfizer), Axitinib, and Sorafenib (BAY43-9006, Nexavar, Bayer) were approved by the FDA as anti-angiogenic agents that directly inhibit the kinase activity of the VEGFR2 intracellular kinase domains (5, 6). Monoclonal antibodies such as bevacizumab (Avastin, Genentech/Roche) and ramucirumab were developed to suppress angiogenesis by binding, respectively, to VEGF and VEGFR2, interrupting VEGFR2 dimerization and consequently inhibiting autophosphorylation (7, 8).

We have developed an antibody–drug conjugate (ADC) approach to suppress angiogenesis. Unlike most of the anti-angiogenic agents that interrupt the kinase signaling cascade by blocking the dimerization of VEGFR2 or by inhibiting kinase activity, our ADC, V21-DOS47, kills VEGFR2-expressing cells by inducing cytotoxic activity at the target cells. Similar to our previous antitumor immunoconjugate, L-DOS47 (9), V21-DOS47 is composed of a camelid single domain antibody and the enzyme urease (derived from jack beans, *Canavalia ensiformis*): the V21 antibody binds to VEGFR2, thus targeting the complex to VEGFR2-expressing cells, whereas the urease enzyme converts endogenous urea into ammonia *in situ* to induce cytotoxicity. Since VEGFR2 is not only expressed in the tumor vasculature but has also been identified on the surface of a variety of tumors (4, 10, 11), V21-DOS47 targets both VEGFR2^+^ vascular endothelial cells and VEGFR2^+^ tumor cells. The elevated local concentration of ammonia also neutralizes the acidic environment surrounding the tumor microvasculature, which is otherwise favorable to cancer cell growth (12). As urease is a plant product with no known mammalian homolog, it is likely to be immunogenic, although an auto-immune reaction is not expected. L-DOS47 is currently being tested in clinical trials and results show that anti-urease antibodies are formed, but no known severe immune toxicity is observed. The full impact of urease immunogenicity is still being studied.

One advantage of camelid single domain antibodies is their relatively small size (approximately 15 kDa) compared to conventional immunoglobulins (approximately 150 kDa). This is particularly important when coupling antibodies to urease, as urease is a large protein with a molecular weight of 544 kDa. By using llama single-domain antibodies, multiple antibodies can be coupled to each urease molecule with a relatively minor increase in overall molecular weight. This allows for the generation of a high avidity therapeutic reagent that retains an acceptable biodistribution profile. Other benefits of single domain camelid antibodies (13–15) are that they are easy to clone and express recombinantly (16, 17), they are generally more thermally and chemically stable than conventional IgG (18, 19), and they bind to epitopes that are not recognized by conventional antibodies (20). In addition, they are not particularly immunogenic as human V_H_ and camelid V_H_H domains share approximately 80% sequence identity (21) and renal clearance is high (22).

Antibody–urease conjugates are complex and large proteins: with multiple antibodies per urease, the molecular weight of the conjugate can reach 680 kDa. This provides a challenge to large-scale production. In our previous report, we described conjugation chemistry and separation procedures designed to address these challenges (9). In this study, we evaluated additional antibody production and conjugation chemistry methods to generate a novel antibody–urease conjugate, V21-DOS47.

In order to produce high-affinity antibodies to VEGFR2, a llama was immunized with recombinant VEGFR2 and a V_H_H phage display library was generated. The V21 antibody was isolated by panning this library with recombinant VEGFR2. Additional amino acid residues were added to the C-terminus of the V21 antibody in order to fulfill multiple objectives: to optimize the antibody pI, to target antibody expression to bacterial inclusion bodies, and to provide a unique target for cross-linking chemistry. In this report, we describe two versions of the V21 antibody, designated V21H1 and V21H4, and the different methods used to conjugate each antibody to urease. Both antibody–urease conjugates were characterized with a variety of analytical techniques, including size exclusion chromatography (SEC) (to evaluate protein purity), SDS-PAGE (to determine the average number of antibodies conjugated per urease), and ESI mass spectrometry (to identify conjugation sites on both the antibody and urease). The effects of conjugation ratio (CR) were examined, and the binding of the two conjugates with the same CR were compared. Binding to VEGFR2 expressed at the cell surface was confirmed by flow cytometry.

## Materials and Methods

### Purification of High Purity Urease (HPU)

Crude urease (cat #U-80, 236 U/mg) was purchased from BioVectra Inc. (Charlottetown, PE, Canada). Prior to use in conjugation, crude urease was purified to remove jack bean matrix protein contaminants such as canavalin and concanavalin A. One million units of crude urease were dissolved in 430 mL of high purity (HP) water at room temperature. The solution was brought to pH 5.15 with 10% (v/v) acetic acid and then centrifuged at 9,000 rcf and 4°C for 40 min. The urease-containing supernatant was cooled to 4°C and fractionated by adding chilled ethanol to a final concentration of 25% (v/v) while maintaining the temperature at 0–8°C. The mixture was stirred overnight and then centrifuged at 9,000 rcf and 4°C for 40 min. The pellet was resuspended in 150 mL of acetate–EDTA buffer (10 mM sodium acetate, 1 mM EDTA, 1 mM TCEP, pH 6.5) and then centrifuged at 4°C and 9,000 rcf for 40 min. The supernatant was concentrated to 75 mL using a Minimate TFF system (Masterflex Model 7518-00 with a Minimate TFF capsule, MWCO 100 kDa), diafiltered three times with 200 mL of acetate–EDTA buffer, and then concentrated down to 100 mL. The diafiltered urease solution was collected, and the strained solution in the capsule and tubing connections was expelled from the system with 50 mL acetate–EDTA buffer and added to the collected solution (total volume ~150 mL). The ethanol fractionated urease solution was further purified by anion exchange chromatography using a Bio-Rad Biologic LP system. The urease solution was loaded at a flow rate of 3.5 mL/min onto a 35 mL DEAE column (DEAE Sepharose Fast Flow, GE Healthcare, cat #17-0709-01) which was pre-equilibrated with 150 mL of IEC Buffer A (20 mM imidazole, 1 mM TCEP, pH 6.5). The column was washed with 100 mL of IEC Buffer A, followed by 80 mL of 40% Buffer B (Buffer A with 0.180 M NaCl). The urease was eluted with 100% Buffer B at a flow rate of 3.5 mL/min and fractions with A_280_ >0.1 were pooled. The pooled fractions were concentrated to a target protein concentration of 6–8 mg/mL using a Minimate capsule with a 100 kDa MWCO membrane and then diafiltered against acetate–EDTA buffer (20 mM sodium acetate, 1 mM EDTA, pH 6.5). The HPU was stored at −80°C. The yield from this purification protocol is typically >55% of the starting activity.

### Expression of V21H1 and V21H4

Both antibodies were expressed in the *E. coli* BL21 (DE3) pT7 system with kanamycin as the selection antibiotic. Transformation of BL21 (DE3) competent *E. coli* cells (Sigma, B2935-10 × 50 µL) was according to the manufacturer’s instructions. One colony from a transformation plate was aseptically inoculated to 200 mL of LB broth (LB media EZ mix, Sigma cat #L76581, 20 g/L) supplemented with 50 mg/L kanamycin. Cultures were incubated at 200 rpm and 37°C. Once the culture reached an OD_600_ greater than 0.6, 50 mL of culture was transferred to four 2 L flasks, each containing 1 L of LB broth with 50 mg/L kanamycin. Flasks were incubated in a shaker incubator at 200 rpm and 37°C. Once the culture reached an OD_600_ of 0.9–1.0, antibody expression was induced by the addition of 1 mM IPTG and overnight incubation at 200 rpm and 37°C. The cells were harvested by centrifugation into aliquots, one per 2 L culture.

### Purification of V21H1

The majority of the V21H1 protein was expressed in the *E. coli* cytosolic solution, not in the inclusion bodies. An aliquot of cell pellet was lysed in 100 mL of lysis buffer (50 mM Tris, 25 mM NaCl, pH 6.5) by sonication in an ice-water bath for 10 min (Misonix 3000 sonicator, tip part #4406; each sonicating cycle: sonicating 30 s, cooling 4 min, power 8). The lysate was centrifuged at 9,000 rcf and 4°C for 30 min. In order to remove the most abundant bacterial matrix proteins, the supernatant was mixed with ice-cold ethanol to a final concentration of 10% (v/v) and incubated in an ice-water bath for 30 min, followed by centrifugation at 9,000 rcf and 4°C for 30 min. The supernatant was mixed with ice-cold ethanol to a final concentration of 45% (v/v) and stirred in an ice-water bath for 60 min, followed by centrifugation at 9,000 rcf and 4°C for 30 min. The pellet was resuspended in 200 mL of wash buffer (50 mM acetate, 0.1% Triton X-100, 1 mM DTT, 25 mM NaCl, pH 5.0). After centrifugation at 9,000 rcf and 4°C for 30 min, the pellet was resuspended in 100 mL of SP Buffer A (50 mM acetate, 8 M urea, pH 4.0) supplemented with 2 mM DTT, and filtered through a 0.45-µm filter. The filtered solution was loaded on to a 1 mL SP FF column (GE Healthcare, cat #17-5054-01) with a peristaltic pump at 2 mL/min, and the column was then connected to an ACTA FPLC system (Amersham Bioscience, cat #UPC-920). After washing the column with 10 mL of SP Buffer A at 1 mL/min, the V21H1 antibody was eluted by a gradient of 0–50% SP Buffer B (SP Buffer A with 0.7 M NaCl) over 30 min at a flow rate of 1 mL/min. The OD_280_ of the peak fraction was determined and the concentration was calculated with an extinction coefficient of 1.967 mg/mL. DTT was added to the SP column peak fraction to a final concentration of 1 mM and the pH of the solution was adjusted to 8–8.5 with 2 M Tris-base. The refolding of the antibody was performed by adding the pH adjusted SP peak fraction drop by drop to refolding buffer (100 mM Tris, 10 µM CuSO_4_, pH 8.8) and continuous stirring at 4°C until the refolding was completed. The refolding process was monitored by intact protein LC-MS. After refolding, the solution was centrifuged at 9,000 rcf and 4°C for 30 min before loading on to a 1 mL QHP column. The column was connected to a FPLC system and washed with 10 mL of Q Buffer A (50 mM HEPES, pH 7.0) at a flow rate of 1 mL/min. The antibody was eluted by a gradient of 0–40% Q Buffer B (Q Buffer A with 0.7 M NaCl) in 40 min at a flow rate of 1 mL/min. The peak fractions from 8 L of cell culture were pooled, concentrated to 2–4 mg/mL, and dialyzed against 20 mM HEPES, pH 7.1 overnight (MWCO 5–8 kDa, volume ratio 1:50) at 4°C. The final V21H1 antibody solution was filtered through a 0.22 µm syringe filter and stored at 4°C.

### Purification of V21H4

In contrast to V21H1, the majority of the V21H4 protein was expressed in the *E. coli* inclusion bodies. The cell pellet from each 2 L culture was resuspended in 100 mL of lysis buffer (50 mM Tris, 25 mM NaCl, pH 6.5) and mixed with lysozyme to a final concentration of 0.2 mg/mL. The cell suspension was incubated at room temperature for 30 min, then lysed by sonication in an ice-water bath for 10 min (Misonix 3000 sonicator, tip part #4406; each sonicating cycle: sonicating 30 s, cooling 4 min, power 8). The lysate was centrifuged at 9,000 rcf and 4°C for 30 min. The pellet was washed twice with 400 mL of Pellet Wash Buffer (50 mM Tris, 25 mM NaCl, pH 6.5, 1% Triton X-100, 2 mM DTT) and once with 50 mM of acetic acid containing 2 mM DTT. The pellet was resuspended in 100 mL of SP Buffer A (50 mM acetate, 8 M urea, pH 4.0) supplemented with 2 mM DTT and centrifuged at 9,000 rcf and 4°C for 30 min. The resulting supernatant was filtered through a 0.45-µm filter and loaded on to a 5 mL SP-XL column (GE Healthcare, cat #17-1152-01) at a flow rate of 5 mL/min. After washing the column with 50 mL of SP Buffer A, the protein was eluted by a gradient of 0–50% SP Buffer B (SP Buffer A with 0.7 M NaCl) over 30 min at a flow rate of 5 mL/min. Peak fractions were collected when A_280_ >700 mU. DTT was added to the pooled SP peak fraction to a final concentration of 1.0 mM and the pH was adjusted to pH 8.6–8.7 with saturated Tris-base. Refolding was initiated by mixing the SP peak fraction with refolding buffer (50 mM Tris, 2 M urea, 1.0 mM DTT, pH 8.6–8.7). After stirring at room temperature for 2 h, 1.2 mM cystamine was added to the refolding mixture. Refolding continued at room temperature and was monitored by RP-HPLC [Agilent 1100 system; ZORBAX-C3 column, PN883750-909; Solvent A: 0.025% (v/v) TFA in water; Solvent B: 0.025% TFA in acetonitrile; gradient: 20–60% B over 30 min at a flow rate of 0.25 mL/min. 100 µL of sample was collected at various time points and acidified by immediately adding 1.0 µL of neat formic acid. 30 µL of each sample was injected to the column to record the chromatogram]. The resulting refolding mixture was centrifuged at 9,000 rcf and 4°C for 30 min before loading to a 5 mL QHP column (GE Healthcare, cat #17-1154-01) at a flow rate of 5 mL/min. After washing the column with 50 mL of Q Buffer A (50 mM HEPES, pH 8.7), the protein was eluted by a gradient of 0–70% Q Buffer B (Q Buffer A with 0.7 M NaCl). Peak fractions with A_280_ >700 mU were pooled. The Q peak fractions were pooled, concentrated to 6–10 mg/mL, and buffer exchanged with 10 mM HEPES, pH 7.1. The final V21H4 antibody solution was filtered through a 0.22-µm filter and stored at 4°C.

### Conjugation of V21H1 to Urease

10 mg of V21H1 antibody was activated with cross-linker at an antibody to cross-linker molar ratio of 1:2.4 by adding 70.4 µL of SM(PEG)_2_ (10.0 mg/mL in DMF) stock solution to the V21H1 antibody while vortexing. The reaction solution was incubated at room temperature for 90 min. The reaction was quenched by adding 300 mM of Tris buffer (pH 7.6) to a final concentration of 10 mM and incubating at room temperature for 10 min. The unconjugated, hydrolyzed, and quenched cross-linker was removed with a 20 mL G25 desalting column pre-equilibrated with 50 mM Tris buffer containing 50 mM NaCl and 1 mM EDTA, pH 7.1. After removing the excess cross-linker, the desalting column fraction was pooled and a 100 µL sample was collected for intact protein mass spectrometric analysis and peptide mapping analysis to evaluate the activation sites on the V21H1 antibody. The remaining pooled fraction was chilled in an ice-water bath for 5 min. 20 mg of HPU was thawed and incubated in another ice-water bath for 5 min. The chilled HPU solution was poured into the activated V21H1 antibody solution while stirring. The stirring continued in an ice-water bath for 5 min, and then the reaction solution was moved to a bench at room temperature. After the conjugation reaction solution was incubated at room temperature for 90 min, cysteine solution (200 mM in 300 mM Tris, pH 7–7.5) was added to a final concentration of 5 mM to quench the reaction. The reaction solution was concentrated down to approximately 4 mL by centrifugation in a 15 mL centrifuge filter (MWCO 100 kDa) at 4°C and 2,000 rcf. The resulting concentrated reaction solution was divided into three aliquots before SEC separation. The separation was performed by loading each aliquot of reaction solution to a Superose 6 100/300 GL column (GE) connected to an AKATA FPLC system. The protein was eluted by an isocratic flow at 0.5 mL/min with SEC buffer (50 mM NaCl, 0.2 mM EDTA, pH 7.2) and the major peak fractions of A_280_ >200 mU were pooled. The peak fractions from all three SEC separations were pooled and dialyzed against 1 L of formulation buffer [10 mM histidine, 1% (w/v) sucrose, 0.2 mM EDTA, pH7.0]. The resulting conjugate solution was filtered through a 0.22-µm filter and divided into 0.8 mL aliquots. Aliquots were stored at −80°C.

### Conjugation of V21H4 to Urease

20 mg of V21H4 was mixed with TCEP (100 mM in 300 mM Tris buffer, pH 7–7.5) to a final concentration of 1.5 mM and incubated at room temperature for 60 min. The excess TCEP and the resulting cysteamine were removed by a 25 mL G25 desalting column using Tris-EDTA buffer (50 mM Tris, 1 mM EDTA, pH 7.1). The resulting desalting fraction was pooled in a 40 mL beaker and diluted with Tris-EDTA buffer to a total volume of 30 mL. The activation reaction was performed by quickly dispensing 0.420 mL of BM(PEG)_2_ stock solution (10 mg/mL in DMF) into the V21H4 antibody solution in the beaker while stirring. After incubation at room temperature for 10 min, the reaction solution was transferred to a 200 mL Amicon diafiltration concentrator with a filter membrane (MWCO 5 kD) and mixed with Tris-EDTA buffer up to 100 mL. The excess cross-linker was removed by connecting the diafiltration concentrator to a 70 psi nitrogen source and concentrated down to 20 mL while stirring. After five cycles of dilution and concentration, the diafiltration concentrator was detached from the nitrogen source and a 100 µL sample was collected to determine the antibody activation sites (using intact protein mass spectrometric analysis and peptide mapping analysis). Tris-EDTA buffer was added to the concentrator to dilute the solution up to the 50 mL marker. The concentrator with the activated V21H4 antibody was chilled in an ice-water bath for 10 min while stirring. After completely thawing at 4°C, 80 mg of HPU was incubated in another ice-water bath for 5 min and then poured into the activated V21H4 antibody solution in the concentrator while stirring in its ice-water bath. After stirring in the ice-water bath for 5 min, the concentrator with the reaction solution was moved to a lab bench and incubated at room temperature for 90 min. The conjugation reaction was quenched by adding cysteine (100 mM in 300 mM Tris, pH 7–7.5) to a final concentration of 5 mM. After quenching the reaction at room temperature for 5 min, the reaction solution was transferred to another container and the concentrator was cleaned and re-installed with a new filtration membrane (MWCO 100 kDa). The reaction solution was transferred back to the concentrator and formulation buffer [10 mM histidine, 1% (w/v) sucrose, and 0.2 mM EDTA, pH 7.0] was added to the 160 mL marker. The concentrator was connected to a 10 psi nitrogen source and concentrated down to 20 mL while stirring. After the dilution–concentration cycle was repeated four times, the diafiltration concentrator was detached from the nitrogen source and the V21H4-DOS47 conjugate solution was transferred to a new container and diluted to 40 mL. The conjugate solution was filtered through a 0.22-µm filter and divided into 0.8 mL aliquots. The aliquots were stored at −80°C.

### Size Exclusion Chromatography

A Waters 2695 HPLC system with a 996 PAD was employed with Empower 2 software for data acquisition and processing. Chromatograms were recorded over 210–400 ± 4 nm with the signal at 280 nm extracted for processing. Separation was performed on a Superose 6 100/300 GL column (GE). Proteins were eluted in 10 mM phosphate, 50 mM NaCl, and 0.2 mM EDTA, pH 7.2. Separation was carried out with an isocratic flow at 0.5 mL/min after injection of a certain volume of neat samples. The column temperature was kept at room temperature, while the sample temperature was controlled at 5 ± 2°C.

### SDS-PAGE

A Bio-Rad Mini Gel Protein Electrophoresis kit and a Bio-RAD Molecular Imager Gel Doc XR+ with ImageLab software were employed to analyze V21-DOS47 CRs. 10 µg of protein samples were mixed with 60 µL of protein gel loading buffer and the mixture was heated to 70°C for 10 min. Denatured samples were loaded (10 µL/well) to a 4–20% Tris–Glycine gel (Invitrogen, cat #XP04200) and electrophoresis was performed at a constant voltage of 150 V with current <40 mA until the electrophoresis front reached the gel bottom. After washing, staining, and destaining, the gel image was scanned with the Gel Doc XR+ imager for analysis. SDS-PAGE was also used to calculate the average number of antibodies conjugated per urease molecule. This was determined by interrogating the intensities of the five bands in the main cluster [see Ref. (9) for further details]. All CRs reported are average values.

### ELISA Assays

A 96-well plate was coated with 100 µL/well of goat anti-human IgG-Fc (Sigma, 5 µg/mL in PBS) at room temperature for 6 h and then blocked with 200 µL/well of 3% BSA/PBS at 2–8°C overnight. After washing 2× with T-TBS (50 mM Tris, 0.15 M NaCl, pH 7.6, containing 0.05% Tween-20), 100 µL/well of VEGFR1/Fc, VEGFR2/Fc, or VEGFR3/Fc [R&D Systems, 0.25 µg/mL in TB-TBS (0.1% BSA/T–TBS)] was added and the plate was incubated at room temperature for 1 h with gentle shaking. After washing 3× with T-TBS, 100 µL/well of antibody–urease conjugate or biotinylated antibody dilutions (in TB-TBS) were added and the plate was incubated at room temperature for 2 h with gentle shaking. For antibody–urease conjugates, plates were washed 3× with T-TBS, 100 µL/well of rabbit anti-urease (1/6,000 or 1/10,000-fold dilution in TB-TBS, Rockland) was added and the plate was incubated at room temperature for 1 h with gentle shaking. For all samples, the plate was washed 3× with T-TBS and 100 µL/well of goat anti-rabbit-AP (1/8,000-fold dilution in TB-TBS, Sigma) was added to detect antibody–urease conjugates or streptavidin–alklaine phosphatase (0.5 µg/mL in TB-TBS, Sigma) was added to detect biotinylated antibodies, and the plate was incubated at room temperature for 1 h with gentle shaking. After washing 3× with T-TBS, 100 µL/well of substrate (4-nitrophenyl phosphate disodium salt hexahydrate, Fluka, 1 mg/mL in diethanolamine substrate buffer, Pierce) was added to each well and incubated at room temperature for 5–15 min with gentle shaking. The absorbance at 405 nm (A_405_) of each well was acquired by scanning the plates with a UV–Vis spectrophotometer.

### Urease Activity Assay

Urease catalyzes the hydrolysis of urea to ammonia. One unit of urease activity is defined as the amount of enzyme that liberates 1 µmol of ammonia per minute at 25°C at pH 7.3. V21H4-DOS47 samples were diluted in sample dilution buffer [0.02 M potassium phosphate containing 1 mM EDTA and 0.1% (w/v) BSA, pH 7.3]. 100 µL of the diluted sample was mixed with 2.00 mL of 0.25 M urea (in phosphate buffer containing 0.3 M sodium phosphate and 0.5 mM EDTA, pH 7.3), and incubated at 25 ± 0.1°C for 5 min, then the reaction was quenched by adding 1.00 mL of 1.0 N HCl. To determine the concentration of ammonium ion produced in the enzyme reaction solution, 100 µL of the quenched reaction solution was mixed with 2.00 mL of phenol solution (0.133 M phenol containing 0.25 mM sodium nitroferricyanide) in a 15 mL testing tube. After 30 s, 2.50 mL of NaOH–NaOCL solution (0.14 N NaOH containing 0.04% sodium hypochlorite) was added to the testing tube, mixed, and incubated at 37°C for 15 min. The absorbance of the solution was determined at 638 nm with the reagent reaction solution (without sample) as the blank. The urease enzyme activity was calculated according to the following equation: U/mL = *D* × (*A* × Tc × Te)/(5 × *E* × Sc × Se) where *A* = absorbance at 638 nm, Tc = total volume of color reaction (4.60 mL), Te = total volume of enzyme reaction (3.10 mL), *E* = molar extinction coefficient of indophenol blue per assay condition (20.10 mM^−1^ cm^−1^), Sc = sample volume for color reaction (0.10 mL), Se = sample volume for enzyme reaction (0.10 mL), and *D* = dilution time. The protein concentration of each sample was determined with a Sigma total protein kit (cat #TP0200) following the manufacturer’s instructions. Urease activity/milligram of conjugate was calculated by dividing the urease activity (units per milliliter) by the amount of protein tested (milligrams per milliliter). Specific urease activity was calculated by dividing the activity/mg conjugate by the proportion of the conjugate’s mass which was composed of urease.

### Western Blot

V21H4-DOS47 test samples and controls were resolved by SDS-PAGE gel electrophoresis and then transferred to a nitrocellulose membrane using a Bio-Rad blot kit. 1.2 µg of HPU and 4.0 µg of V21H4 as controls and 2.0 µg of V21H4-DOS47 samples were mixed with 60.0 µL of protein gel loading buffer. The resulting sample mixtures were denatured by heating to 60°C for 10 min and 10 µL of each sample was loaded per lane. Duplicate blots were made from gels run in parallel for urease and V21H4 antibody probing. For urease detection, a rabbit anti-urease IgG (Rockland) was used. To detect the V21H4 antibody, a rabbit anti-llama IgG (ImmunoReagents Inc.) was used. A goat anti-rabbit IgG conjugated to AP (Sigma) was used as the secondary visualization antibody. Final development of the Western blots was performed with AP buffer containing NBT/BCIP.

### Mass Spectrometry

A Waters Xevo G2 QTOF mass spectrometer and an Acquity UPLC system H class were employed for all mass spectrometry analyses. A lock mass of 785.8426 Da was applied for real time point to point mass calibration. LC-MS data acquisition was controlled by Masslynx V4.1 software.

### Intact Protein Mass Spectrometry Analyses

Cross-linker-activated antibody samples were reacted with 5 mM cysteine at room temperature for 30 min, diluted to 0.5–1 mg/mL in water, and acidified by adding neat formic acid to a final concentration of 1% (v/v). A BEH300 C4 (1.7 µm, 2.1 × 50 mm) column was used. The column temperature was set at 60°C and Solvent A (0.025% v/v TFA in water) and Solvent B (0.025% TFA in acetonitrile) were used for UPLC separation. The UPLC was performed with a flow rate of 0.15 mL/min with a gradient from 20 to 60% Solvent B over 30 min. LC-MS TIC (total ion counts) data acquisition was carried out in an *m/z* range of 500–3,500 Da in resolution mode with a scan rate of 0.3/s, capillary voltage 3.0 kV, sample cone voltage 40 V, extraction cone voltage 4.0 kV. Ion source temperature was set at 100°C and desolvation temperature was set at 350°C. Desolvation gas flow rate was 600 L/h. A real time lock mass TIC raw data set (scan/20 s) was acquired with 100 fmol/μL Glu-Fib B at a flow rate of 6.0 µL/min. Mass spectrometric raw data were processed with BiopharmaLynx software (v 1.2) in intact protein mode with a resolution of 10,000. Mass match tolerance was set at 30 ppm, and the protein sequence of each antibody containing one disulfide bond was input as the match protein for protein match searches.

### Tryptic Digestion of V21H1-SM(PEG)_2_-Cys and V21H4-BM(PEG)_2_-Cys

The cross-linker-activated antibody samples were reacted with 10 mM cysteine at room temperature for 30 min and then diluted to 0.5 mg/mL with 100 mM ammonia hydrogen carbonate. Neat acetonitrile was added to the diluted sample solution to a final concentration of 20% (v/v). Trypsin/Lys-C mix (Promega, cat #V507A) was added at a protein:protease ratio of 20:1 and digested at 37°C for 16–20 h. DTT was added to the digested sample to a final concentration of 10 mM and samples were incubated at 37°C for 30 min to reduce the core disulfide bond. The digestion was stopped by adding neat formic acid to 1% (v/v) before mass spectrometry analysis.

### Tryptic Digestion of V21H4-DOS47

100 µg of V21H4-DOS47 was mixed with DTT to a final concentration of 10 mM and neat acetonitrile was added to a final concentration of 20% (v/v). To reduce the disulfide bond and denature the conjugated proteins, the sample mixture was heated at 60°C for 30 min. The denatured protein precipitate was pelleted by centrifugation at 16,000 rcf at room temperature for 5 min. 5.0 µL of 0.20 M iodoacetamide and 100 µL of water were added to the pellet then mixed by vortexing. The suspension was centrifuged at 16,000 rcf at room temperature for 5 min and the supernatant was discarded. The resulting pellet was dissolved in 100 µL of Tris–guanidine buffer (4 M guanidine chloride, 50 mM Tris, 10 mM CaCl_2_, and 10 mM iodoacetamide, pH 8.0). After this alkylation reaction was performed at room temperature in the dark for 30 min, the reaction was quenched with 5 mM DTT. The resulting solution was diluted four times with Tris buffer (50 mM Tris, 10 mM CaCl_2_, pH 8.0). Trypsin/Lys-C mix was added to the diluted sample solution at a protein:protease ratio of 25:1. After the digestion was performed at 37°C for 16–20 h, the reaction was stopped by adding neat formic acid at a final concentration of 1% (v/v).

### LC-MS^E^ Peptide Mapping of V21H1-SM(PEG)_2_-Cys, V21H4-BM(PEG)_2_-Cys, and V21H4-DOS47 Tryptic Digests

A BEH300 C18 (1.7 µm, 2.1 mm × 150 mm) column was used for UPLC separation. The column temperature was set at 60°C. Solvent A (0.075% v/v formic acid in water) and Solvent B (0.075% formic acid in acetonitrile) were used for peptide elution. UPLC was performed with a flow rate of 0.15 mL/min. A gradient of 0–30% Solvent B in 50 min was used for the separation of the tryptic digests of V21H1-SM(PEG)_2_-Cys and V21H4-BM(PEG)_2_-Cys samples. For the tryptic digests of V21H4-DOS47, a gradient of 0–45% Solvent B in 150 min was used. LC-MS^E^ TIC (total ion counts) data acquisitions were carried out in an *m/z* range of 50–2,000 Da in resolution mode with a scan rate of 0.3/s, capillary voltage 3.0 kV, sample cone voltage 25 V, and extraction cone voltage 4.0 kV. Ion source temperature was set at 100°C and desolvation temperature was set at 350°C. Desolvation gas flow rate was 600 L/h. A real time lock mass TIC raw data set (scan/20 s) was acquired with 100 fmol/μL Glu-Fib B at a flow rate of 3.0 µL/min. With the instrument setup, two interleaved scan functions are applied for data acquisitions. The first scan function acquires MS spectra of intact peptide ions in the sample while applying no energy to the collision cell. The second scan function acquires data over the same mass range; however, the collision energy is ramped from 20 to 60 eV. This scan is equivalent to a non-selective tandem mass spectrometric (MS/MS) scan and allows for the collection of MS^E^ fragment spectra from the ions in the preceding scan. The high energy collision induced fragmentation randomly cleaves peptide backbone bonds. For each C-N peptide backbone bond cleaved, the amino-terminal ion generated is called the “*b*” ion and the C-terminal ion generated is called the “*y*” ion. In Tables 1–3, the column entitled “MS/MS *b*/*y* Possible” indicates the theoretical maximum number of *b* and *y* ions that would be produced for each peptide if all peptide bonds in the protein were equally likely to be broken. The column entitled “MS/MS *b*/*y* Found” indicates the actual number of *b* and *y* ions identified for each peptide. The identification of *b*/*y* ions provides unambiguous confirmation of peptide identity. Mass spectrometric raw data were processed with BiopharmaLynx software (v 1.2) in peptide map mode with a resolution of 20,000. A lock mass of 785.8426 Da was applied for real time point to point mass calibration. The low energy MS ion intensity threshold was set at 3,000 counts and the MS^E^ high energy ion intensity threshold was set at 300 counts. Mass match tolerances were set at 10 ppm for MS and at 20 ppm for MS^E^ data sets. Peptides with one missed cleavage site were included in mass match searching. V21H1, V21H4, and urease (Uniprot P07374) protein sequences were, respectively, input into the sequence library for peptide matching/identification. Variable modifiers, including deamidation N, deamidation succinimide N, oxidation M, +K, +Na, and carbamidomethyl C (for alkylated cysteine) were applied for peptide map analysis. SM(PEG)_2_-Cys (429.1206 Da) was set as a variable modifier to identify the activation sites of V21H1 conjugation, whereas BM(PEG)_2_-Cys (431.1362 Da) was input as a variable modifier to identify the activation sites of V21H4 conjugation. For the V21H4-DOS47 tryptic digests, GGGEEDDGC-BM(PEG)_2_ (1,145.3453 Da) was set as a variable modifier to identify the conjugation sites on urease.

**Table 1:** List of identified peptides and activation sites of V21H1-(PEG)_2_-Cys.

Tryptic peptide	Activation site	Calculated mass (Da)	MS/MS *b*/*y* possible	MS/MS *b*/*y* found	Intensity	Mass match error (ppm)	% of activation
T001		1,985.0364	38	37	28847130	−2.4	
T001*	M_1_-SM(PEG)_2_-Cys	2,416.1726	38	32	5688300	0.2	15.7
T001–002		2,730.3904	54	28	1681792	0.8	

T002		763.3647	14	9	14953790	0.9	
T002–003		2,066.9456	36	2	16053	2.8	
T003		1,321.5913	20	18	87904800	−2.4	
T003–004		180.8562	30	nd	nd	nd	

T004		499.2754	8	7	238539	0.4	
T004–005		784.4191	12	9	1334242	1.1	
T004–005*	K_44_-SM(PEG)_2_-Cys	1,215.5553	12	6	369637	0.9	18.4
T005		303.1543	2	0	61996	−3	

T005–006		2,503.1868	42	28	5351105	−1.2	

T006		2,218.043	38	33	20205530	−0.2	
T006–007		2,431.1655	42	10	203405	−3	12.4
T006–007*	K_66_-SM(PEG)_2_-Cys	2,862.3018	42	29	2900557	−0.1	
T007		231.1331	2	1	147694	−2.2	

T007–008		835.4664	12	9	1990138	−2.8	
T008		622.3439	8	6	19702980	0	
T008–009		1,050.5458	16	11	747025	0.1	

T009		446.2125	6	5	269841	−1.3	
T009–010		3,129.49	54	nd	nd	nd	
T009–010*	K_77_-SM(PEG)_2_-Cys	3,560.6262	54	31	4111249	1.9	3.6
T010		2,701.2881	46	36	108301696	−2.7	

T010		2,701.2881	46	36	108301696	−2.7	
T010*	K_88_-SM(PEG)_2_-Cys	3,132.4241	46	23	1836133	−0.8	1.7

T010–011		6,145.7744	108	nd	nd	nd	
T010–011	K_101_-SM(PEG)_2_-Cys	6,576.9103	60	nd	nd	nd	

T011		3,462.4971	60	7	105092	1.9	
T011–012		3,590.592	62	43	48704060	0.1	
T011–012*	K_131_-SM(PEG)_2_-Cys	4,021.7283	62	12	258662	−1.7	0.5

T012		146.1055	0	nd	nd	nd	
T012	K_132_-SM(PEG)_2_-Cys	577.2418	0	nd	nd	nd	

*Thick boxes (also shaded blue) around sets of tryptic peptides indicate related groups of peptides used to calculate percentage of activation for each activation site*.

*nd, not detected*.

*indicates the modified version of the peptide.

**Table 2:** List of identified peptides and activation sites of V21H4-(PEG)_2_-Cys.

Tryptic peptide	Activation site	Calculated mass (Da)	MS/MS *b*/*y* possible	MS/MS *b*/*y* found	Intensity	Mass match error (ppm)	% of activation
T001		1,985.0364	38	34	25539260	1.2	
T001–002		2,730.3904	54	17	55292	0.6	

T002		763.3647	14	8	7457241	−0.7	
T002*	C_23_-BM(PEG)_2_-Cys	1,192.4852	14	5	169047	2.1	2.2
T002–003		2,066.9456	36	nd	nd	nd	

T003		1,321.5913	20	18	29459300	−0.5	
T003–004		1,802.8562	30	nd	nd	nd	
T004		499.2754	8	5	254649	−2.6	
T004–005		784.4191	12	8	1083205	−2.7	
T005		303.1543	2	1	69756	−4	
T005–006		2,503.1868	42	27	4016949	3	
T006		2,218.043	38	35	10074250	−0.4	
T006–007		2,431.1655	42	2	57264	4	
T007		231.1331	2	1	168759	−4.3	
T007–008		835.4664	12	10	1210281	−2.9	
T008–009		1,050.5458	16	6	92188	−2.4	
T009		446.2125	6	5	247926	−0.9	

T009–010		3,129.49	54	nd	nd	nd	
T010		2,701.2881	46	35	62124531	1.3	
T010*	C_97_-BM(PEG)_2_-Cys	3,130.4087	46	7	334626	3.3	0.5
T010–011		5,613.6455	98	nd	nd	nd	

T011		2,930.3682	50	37	18549570	−1.4	

T011–012		3,749.6023	68	nd	nd	nd	
T012		837.2446	16	8	150911	−2.1	
T012*	C_136_-BM(PEG)_2_-Cys	1,266.3652	16	10	1885506	−0.2	92.6

*Thick boxes (also shaded blue) around sets of tryptic peptides indicate related groups of peptides used to calculate percentage of activation for each activation site*.

*nd, not detected*.

*indicates the modified version of the peptide.

**Table 3:** ESI LC-MS^E^ peptide mapping analysis: Identification of urease cysteine residues modified by V21H4-(PEG)_2_-Cys.

Conjugation sites searched from the urease side							

Urease peptide	Conjugation site	Calculated mass (Da)	MS/MS *b*/*y* Possible	MS/MS *b*/*y* found	Intensity	Mass match error (ppm)	% of conjugation
1:T010*	UC_59_-VC_136_	2,784.2053	28	10	335045	2.6	2.6
1:T026*	UC_207_-VC_136_	1,939.6624	12	0	10296	1.9	0.6
1:T063*	UC_663_-VC_136_	2,316.7554	18	4	46812	2.9	4.2
1:T081*	UC_824_-VC_136_	2,633.1372	26	13	495879	2.1	26.7

**Conjugation sites searched from the antibody side**							

**V21H4 C-term peptide**	**Conjugation site**	**Calculated mass (Da)**	**MS/MS *b*/*y* possible**	**MS/MS *b*/*y* found**	**Intensity**	**Mass match error (ppm)**	**% of conjugation**

2:T012	na	837.2446	16	2	10403	−3.9	0.4
2:T012*	-UC_824_	2,633.1472	16	7	1609854	1.2	59.1
2:T012*	-UC_663_	2,784.2153	16	5	726682	1.6	26.7
2:T012*	-UC_59_	2,316.7654	16	4	343529	−1.4	12.6
2:T012*	-UC_207_	1,939.6724	16	0	33038	−3.6	1.2

*na, not applicable*.

*indicates the modified version of the peptide.

### Flow Cytometry

293 or 293/KDR cells were detached from flasks using non-enzymatic cell dissociation buffer (Sigma). Cells were centrifuged at 300 × *g* for 5 min and then resuspended in staining buffer at 10^6^ cells/mL (PBS with Ca^2+^ and Mg^2+^, 0.02% NaN_3_, 2% FBS). 100 µL of cells was added to wells of a 96-well plate. The plate was centrifuged at 350 × *g* for 4 min, buffer removed, and then cells were resuspended in 50 µL of antibody–urease conjugate or biotinylated antibody (diluted in staining buffer) and then incubated at 2–8°C for 1 h. For cells stained with antibody–urease conjugates, cells were washed 3× with staining buffer and then resuspended in mouse anti-urease (Sigma, cat #U-4879) at 5.8 µg/mL (diluted in staining buffer) and incubated for 30 min at 2–8°C. For all samples, cells were washed 3× with staining buffer and then resuspended in AF488-anti-mouse IgG (Jackson, cat #115-545-164) at 3 µg/mL (diluted in staining buffer) for antibody–urease samples or with PE-SA (Biolegend, cat #405204) at 133 ng/mL (diluted in staining buffer) for biotinylated antibodies. All cells were incubated for 30 min at 2–8°C in the dark, washed 3× with staining buffer, then resuspended in 1% paraformaldehyde (diluted in PBS). The plate was incubated for 15 min at room temperature, covered with tin foil. The plate was then centrifuged as above, paraformaldehyde removed, and the cells were resuspended in staining buffer. The plate was covered in tin foil and stored at 2–8°C until analysis using a Guava flow cytometer and guavaSoft software (Millipore). S/N values are the ratio of V21H4-DOS47 binding to 293/KDR cells vs V21H4-DOS47 binding to 293 cells or the ratio of biotin-V21H4 vs biotin-isotype control antibody (anti-CEACAM6) binding to 293/KDR cells.

## Results

### Production and Purification of V21H1

When generating single domain antibodies for immunoconjugate drugs, HP antibodies must be produced at high yield and with controllable processes, including expression, protein refolding, and purification. Other considerations include the following: the pI of the antibody should be such that the antibody conjugate is stable and soluble at physiologic pH, the properties of the antibody should be suitable for the conjugation chemistry, and the modifications of the antibody residues during conjugation reactions should not compromise the affinity of the antibody binding to its antigen.

**Figure 1:**
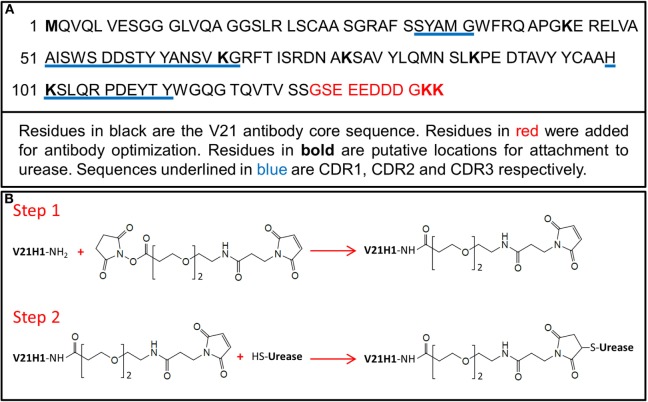
**(A)** Amino acid sequence of the V21H1 antibody. **(B)** Description of conjugation strategy used to conjugate V21H1 to urease to generate V21H1-DOS47. Step 1 is the activation of the antibody using SM(PEG)_2_. Step 2 conjugates the activated antibody to urease.

The V21 camelid antibody consists of 122 amino acids (ending at S_122_, Figure 1A). Eleven amino acids were added to the C-terminus of the V21 antibody in order to generate V21H1. By adding these amino acids, the pI of the antibody was changed from 8.75 to 5.44, as required for conjugate stability and solubility. The heterobifunctional chemical cross-linker SM(PEG)_2_ reacts with amine and sulfhydryl groups and was selected for use in conjugating V21H1 to urease (Figure 1B). There are five lysine residues in the core V21 sequence, two of which (Lys_66_ and Lys_101_) are located in the CDR2 and CDR3 sequences, respectively. As these amino acids could be modified by the amine conjugation chemistry utilized by SM(PEG)_2_, potentially altering antibody activity, two extra lysine residues were added to the antibody C–terminus to minimize this probability.

V21H1 was expressed primarily in the cytosolic solution of BL21(DE3) bacteria, with virtually no expression in inclusion bodies. Therefore, after cell lysis, the antibody was separated from bacterial proteins by ethanol crystallization and cation exchange chromatography. After antibody refolding, the native antibody was further purified by anion exchange chromatography. To confirm that the molecular mass of the purified antibody matched the designed protein sequence, LC-MS intact protein analysis was performed. No impurity proteins were detected from the LC-MS TIC chromatograms and the detected molecular mass of V21H1 matched the theoretical value calculated from its protein sequence within 30 ppm mass match error (data not shown). However, the yield of purified V21H1 was very low (4–6 mg/L of culture) and the purification processes used are not suitable for large-scale cGMP production.

### Cross-Linker Activation of V21H1

V21H1 was activated by SM(PEG)_2_ at pH 7.0 using conditions previously found to be optimal for activation of AFAIKL2 antibody with SIAB in the production of the antibody–urease conjugate L-DOS47. Since the NHS-ester reaction is the same for SIAB and SM(PEG)_2_ and the LC-MS spectra are similar for AFAIKL2 and V21H1 reaction products (data not shown), these conditions should also be optimal for activation of V21H1 with SM(PEG)_2_.

**Figure 2:**
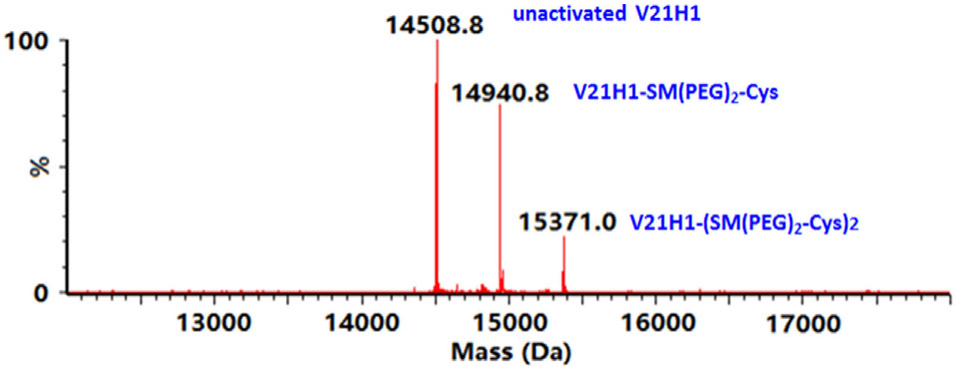
Deconvoluted mass spectrum of the V21H1 antibody after activation by cross-linker and linkage to cysteine showing the distribution of non-activated antibody, antibody activated by one cross-linker, and antibody activated by two cross-linkers.

Only the NHS-ester group of SM(PEG)_2_ can react with V21H1. The two cysteine residues in the V21H1 antibody form a disulfide bond and are, thus, unavailable to react with the maleimido end of the cross-linker. The primary amines from the antibody N-terminus and the lysine residues from the protein sequence can all potentially react with the NHS-ester of the cross-linker. The maleimido end of the antibody-carrying cross-linker then reacts with cysteines on the surface of urease molecules. The probability of each amine being activated depends on its accessibility due to its surrounding native structure. To avoid urease dimer and polymers forming in the second reaction step, ideally only one amine per antibody would be activated by the NHS-ester. However, since multiple primary amines are present in each antibody, it is statistically inevitable that some V21H1 antibodies will be activated by more than one cross-linker molecule. The optimal activation condition was selected, which minimizes the percentage of antibodies that are activated by more than one cross-linker while maximizing the total amount of activated antibody. To assess the activation distribution, the SM(PEG)_2_ activated V21H1 was reacted with excess cysteine and evaluated by intact mass spectrometric analysis. The mass spectrum is shown in Figure 2. Approximately 50% of the V21H1 was activated by SM(PEG)_2_ and of the activated antibody, approximately 30% was activated by two cross-linkers. Thus, only 35% of the V21H1 antibody is optimally activated for cross-linking with urease.

In order to determine which lysines of V21H1 were targeted by SM(PEG)_2_, V21H1-SM(PEG)_2_-Cys was subjected to tryptic digestion followed by LC-MS^E^ analysis. Trypsin cleaves peptide backbone bonds at the C-terminal side of arginine and lysine residues (unless proline is immediately C-terminal to K or R). If a lysine is activated by SM(PEG)_2_, the polarity and side-chain structure of the lysine is altered and spatially blocked. Thus, this tryptic site is no longer accessible to the protease. For example, if K_66_ of V21H1 is activated by SM(PEG)_2_, it is linked to -SM(PEG)_2_-Cys and is no longer available for tryptic digestion; therefore, a peak with a molecular mass of 2,862.3018 (2,431.1656 + 431.1362) Da should be observed, which represents the -SM(PEG)_2_-Cys linked lysine-in-middle peptide (ELVAAISWSDDSTYYANSVK_66_GR)-SM(PEG)_2_-Cys. In the LC-MS^E^ peptide mapping analysis, all possible activation sites can be identified by searching all the lysine carrying peptides and the N-terminal peptide with the -SM(PEG)_2_-Cys (431.1362 Da) as a variable modifier. The detected tryptic peptides along with conjugation sites are listed in Table 1.

All tryptic peptides were detected with mass match errors of less than 5 ppm and the amino acid sequence recovery was 100%. Assuming that ESI sensitivity is not affected by the linkage of the modifier, an activation percentage was assessed by comparing the intensity of the cross-linker modified peptide with the sum intensity of all the related peptides. Under the activation conditions used, lysine residue K_66_ in CDR2 was substantially (~25% of the entire activated V21H1 antibody) activated by the cross-linker; however, K_101_ in CDR3 was not modified during cross-linker activation. Surprisingly, the two C-terminal lysine residues that were intentionally added for conjugation chemistry purposes were not modified by the cross-linker.

### Production and Purification of V21H4

**Figure 3:**
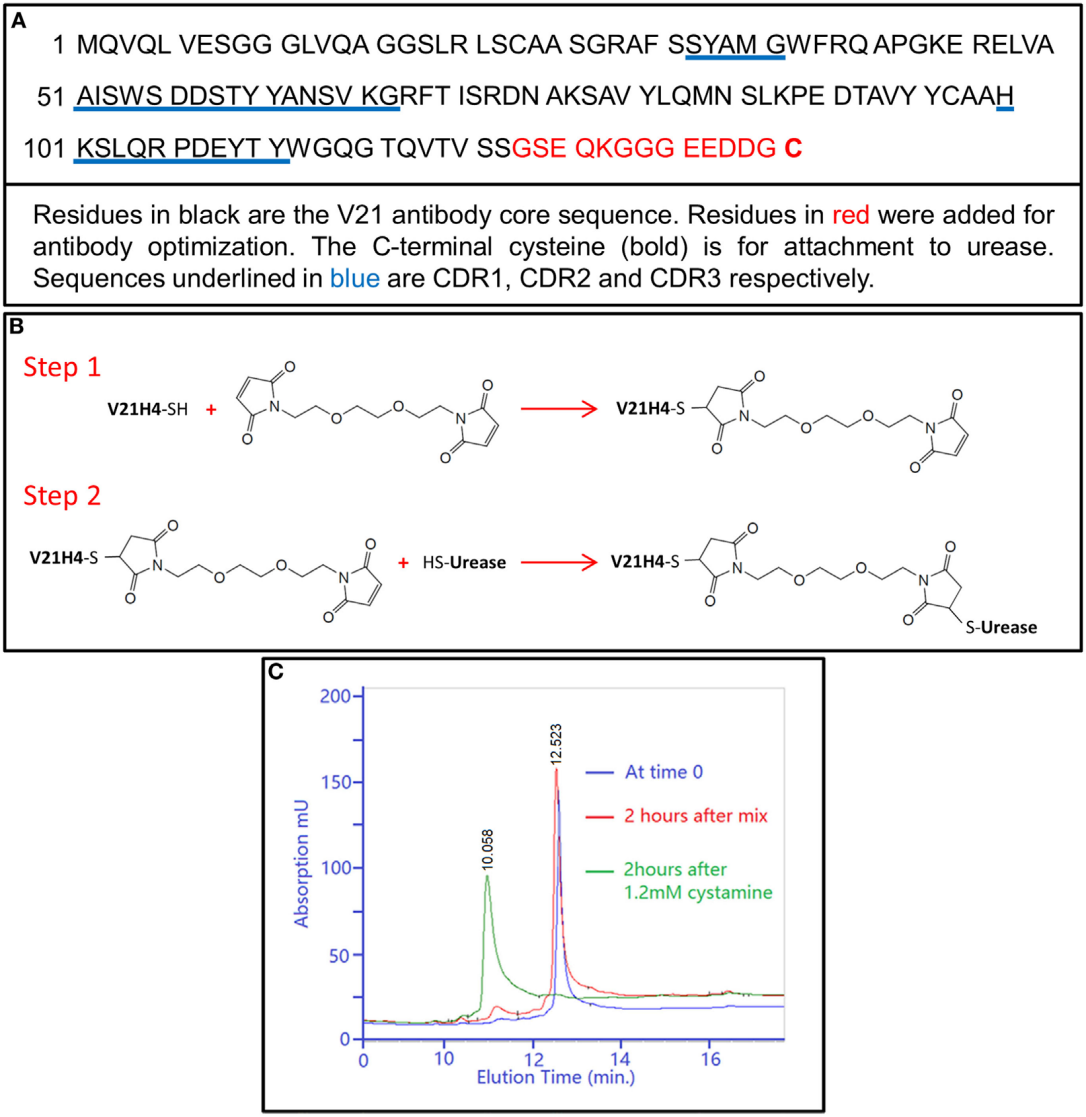
**(A)** Amino acid sequence of the V21H4 antibody. **(B)** Description of conjugation strategy used to conjugate V21H4 to urease to generate V21H4-DOS47. Step 1 is the activation of the antibody using BM(PEG)_2_. Step 2 conjugates the activated antibody to urease. **(C)** RP-HPLC chromatograms of V21H4 samples at different refolding time points. Blue line: sample at refolding time 0, immediately after the SP pooled fraction was mixed with refolding buffer. Red line: refolding time point 2 h after mixing. Green line: refolding sample 4 h after time 0 and 2 h after addition of 1.2 mM cystamine. Unfolded antibody elutes at 12.513 min and folded antibody elutes at 10.958 min.

The antibody V21H4 was designed to improve upon the issues identified during production, purification, and cross-linker activation of V21H1. The amino acid sequence of the V21H4 antibody is shown in Figure 3A. As for V21H1, a number of amino acid residues were added to the antibody C-terminus (G_123_–C_136_) and the pI of the antibody was adjusted from 8.75 to 5.43. With V21H1, the presence of SM(PEG)_2_ cross-linker activated K_66_ in the antibody CDR2 region was a concern as this could impair antibody binding affinity. Thus, a cysteine residue (C_136_) was added to V21H4 for sulfhydryl-to-sulfhydryl cross-linking using a different cross-linker, BM(PEG)_2_ (Figure 3B). The inclusion of a C-terminal cysteine also allowed the antibody to be expressed in bacterial inclusion bodies. As the two core cysteine residues of the V21 antibody form a disulfide bond and are unavailable for chemical conjugation, the additional C-terminal cysteine residue provides a unique activation site for targeted conjugation.

As expected, V21H4 was expressed at high levels in inclusion bodies. After cell lysis, antibody was separated from bacterial matrix proteins by centrifugation. The denatured antibody was purified by cation exchange chromatography to remove nucleic acids and other proteins. The refolding of the V21H4 antibody was performed in an easily controllable manner and was monitored by HPLC (Figure 3C).

The refolding process was initiated by mixing the peak fraction of the cation exchange column with refolding buffer. While the folding process was very slow without cystamine, folding was complete in 2 h at room temperature after cystamine was added to a final concentration of 1.2 mM. Anion exchange chromatography was used to isolate the properly folded protein and yields of greater than 80% were generally observed. The typical yield of purified V21H4 is 20–40 mg/L culture, which is considerably higher than that of V21H1. In addition, the method used to produce and purify V21H4 is amenable to scale up and cGMP procedures.

### Cross-Linker Activation of V21H4

**Figure 4:**
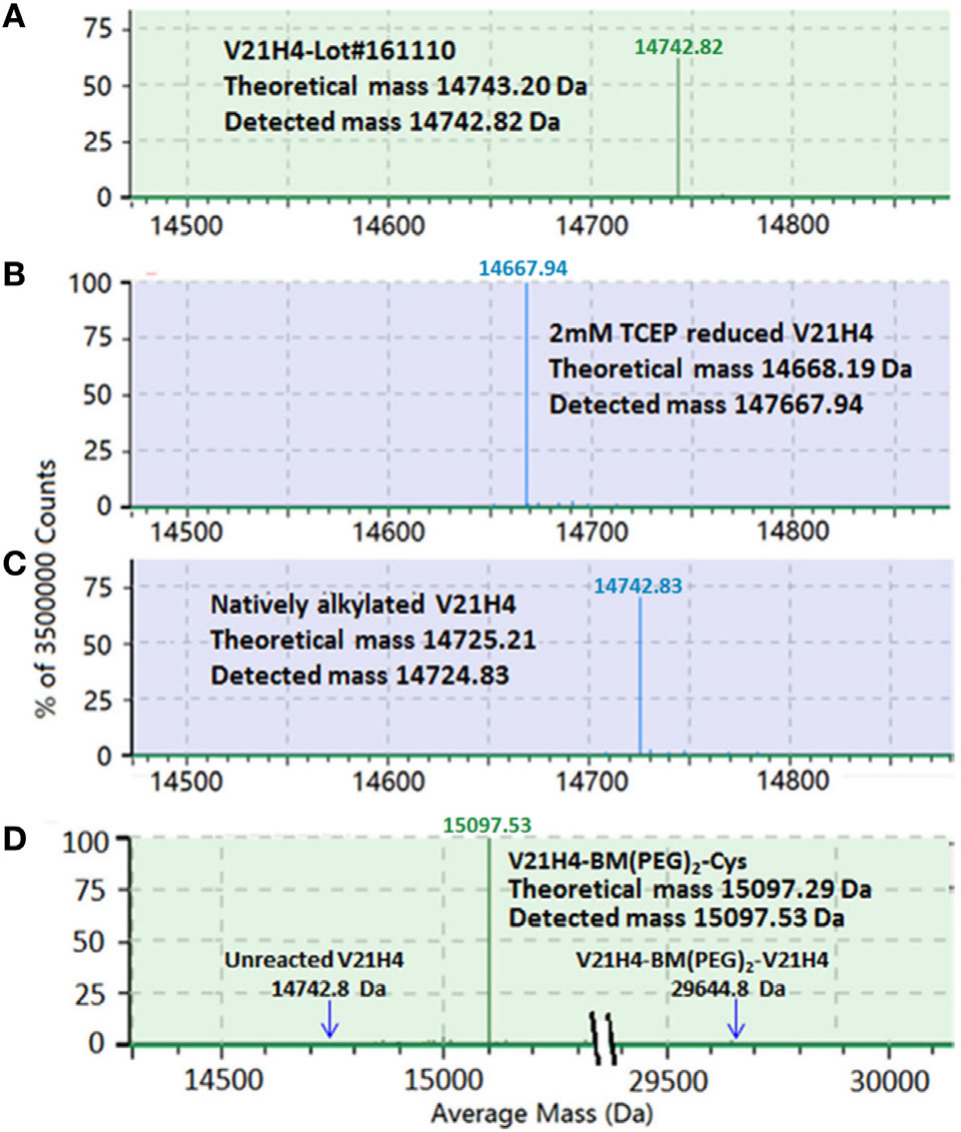
**(A–D)** Screen snapshots of intact protein mass spectra of V21H4 samples from BiopharmaLynx. **(A)** Deconvoluted spectrum of V21H4 showing the attachment of a half cystamine to the C-terminal cysteine by forming a disulfide bond during refolding. **(B)** The deconvoluted spectrum of V21H4 after reduction with 2 mM TCEP showing the detachment of the C-terminal half cystamine. **(C)** The deconvoluted spectrum of the reduced V21H4 after alkylation with iodoacteamide showing the C-terminal cysteine is accessible to a sulfhydryl activation cross-linker. **(D)** Deconvoluted mass spectrum of V21H4 after activation by cross-linker and linkage to cysteine. V21H4 antibody activated by BM(PEG)_2_ generates a single activated species.

The C-terminal cysteine of V21H4 is required for conjugation to urease. However, as cystamine was included in the V21H4 refolding buffer, the C-terminal cysteine was modified by forming a disulfide bond with a half cystamine (cysteamine-H). This was confirmed by LC-MS intact protein analysis (Figure 4A). Thus, the half cystamine must be removed and the cysteine must subsequently be available for activation by cross-linker. In addition, this removal must occur using a controllable mild reduction under the native conditions to be used for conjugation purposes and it must not reduce the antibody’s internal disulfide bond. As shown in Figure 4B, after reducing V21H4 with 2 mM TCEP at pH 7.1 for 1 h at room temperature, the detected antibody molecular mass was 14,667.94 Da, suggesting that the protective half cystamine had been removed. In order to confirm that the de-protected cysteine residue was active to the cross-linking reagent, 10 mM iodoacetamide was added to the de-protected V21H4 antibody. After 30 min at room temperature at pH 7.5–8.0, the resulting detected molecular mass was increased to 14,724.83 Da (Figure 4C), suggesting a carboxymethyl group (57.05 Da) was alkylated to the cysteine residue. In summary, the C-terminal half cystamine can be removed and the resulting de-protected cysteine is available for chemical conjugation. The alkylated antibody was also digested with trypsin and evaluated by LC-MS^E^ peptide mapping. The LC-MS^E^ peptide map (data not shown) covered 100% of the amino acid sequence and the C-terminal cysteine was specifically and effectively alkylated, confirming the specificity of the de-protective reduction reaction and the suitability of the C-terminal cysteine in targeted sulfhydryl cross-linking chemistry.

The V21H4 antibody was activated by the cross-linker BM(PEG)_2_. As BM(PEG)_2_ is a homobifunctional cross-linker, it is possible that both maleimido groups of BM(PEG)_2_ could react with and link two V21H4 molecules, leading to the generation of antibody dimers that cannot conjugate to urease. The frequency of antibody dimers generated depends upon the molar ratio of the reactants, the native hydrophobicity environment of the cysteine residue and the relative mobility of the molecules in the reaction solution. This reaction was performed with a 10:1 cross-linker to antibody molar ratio. In addition, the molecular weight of the cross-linker is 308.29 Da, which is approximately 50-fold less than the molecular weight of the antibody. To evaluate the activated V21H4 antibody, 100 µL of the activated antibody solution was reacted with excess cysteine and evaluated by intact mass spectrometric analysis (Figure 4D). Under the experimental conditions used, more than 99% of the V21H4 was coupled to a single cross-linker, leaving the cross-linker’s other maleimido group available for the subsequent reaction to urease.

In order to confirm that the C-terminal cysteine was the sole target of BM(PEG)_2_, V21H4-BM(PEG)_2_-Cys was subjected to tryptic digestion followed by LC-MS^E^ analysis. If the C-terminal cysteine is activated by the cross-linker, a peak with a mass of 1,266.3652 Da representing the cross-linker activated peptide GGGEEDDGC_136_-BM(PEG)_2_-Cys should be detected. If the core disulfide bond is reduced by TCEP before cross-linker activation, then two peaks—one representing the peptide LSC_23_AASGR-BM(PEG)_2_-Cys (1,192.4852 Da) and the other representing SAVYLQMNSLKPEDTAVYYC_97_AAHK-BM(PEG)_2_-Cys (3,130.4087 Da) should be identified. The detected tryptic peptides along with the cross-linker activation sites are listed in Table 2. All tryptic peptides were detected with mass match errors of less than 5 ppm, and the amino acid sequence recovery was 100%. As expected, more than 90% of the C-terminal cysteine was activated by the cross-linker, and only trace amounts of cross-linker activated core cysteine residues (Cys_23_ and Cys_97_) were detected. This is a much more desirable scenario than that observed with V21H1 and SM(PEG)_2_, in which multiple lysines were targeted, including the one in CDR2.

### Conjugation of V21H1 and V21H4 to Urease and Initial Characterization

Jack bean urease is a homohexameric enzyme with each subunit approximately 91 kDa. Among the 15 unbound cysteine residues per subunit, five are on the surface of the native structure and are available for linking to single domain antibodies through maleimido cross-linkers (23). Different conjugation chemistries are widely used for protein conjugations. Copper-free click chemistry has been preferentially used in protein labeling and protein–drug conjugations (24) and was a potential option in our conjugations of antibodies to urease. However, either the NHS-ester or maleimido activation step would be needed before performing the click chemistry. Thus, traditional cross-linking chemistries are simpler and are suitable to this particular case.

**Figure 5:**
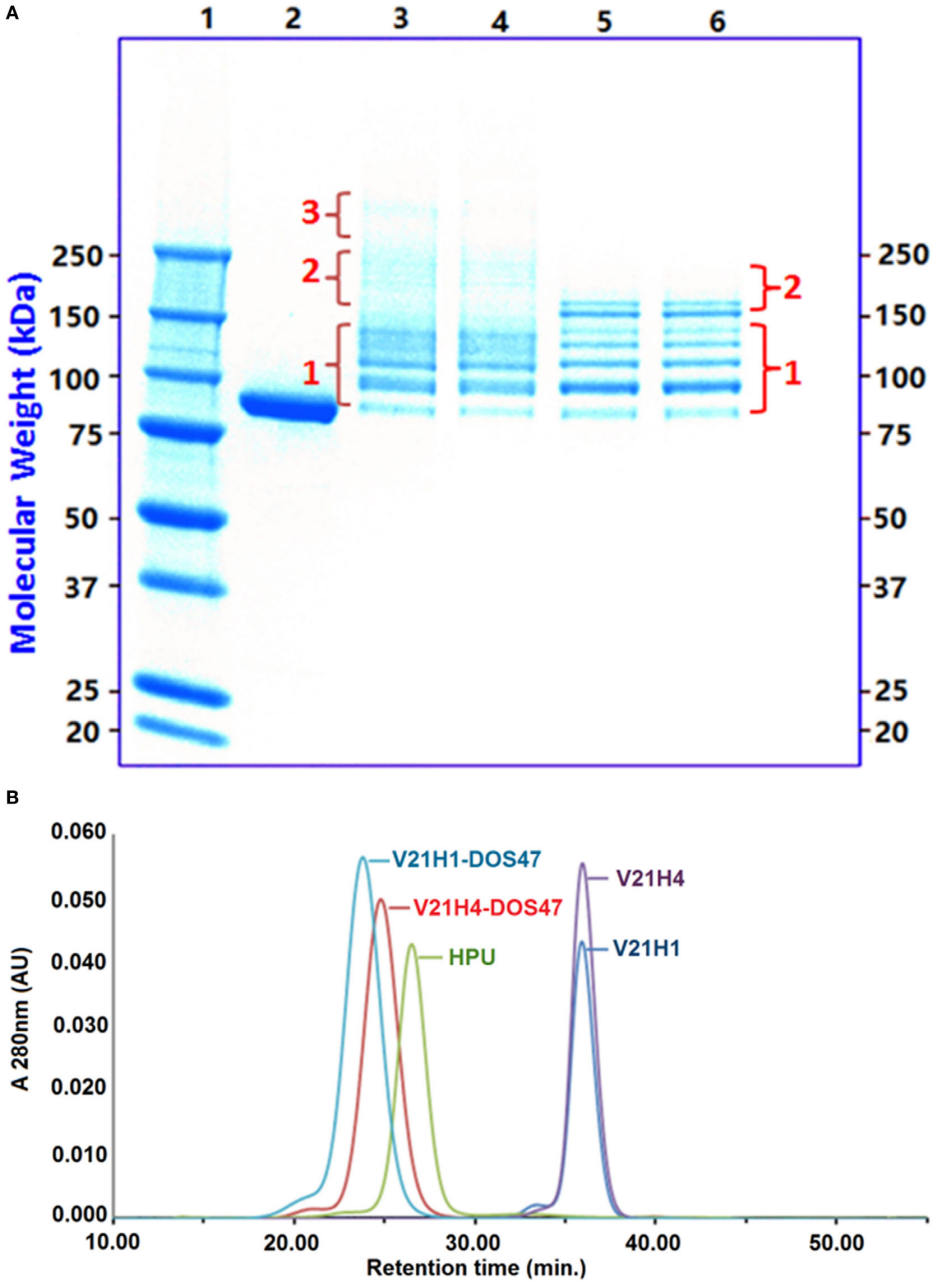
**(A)** SDS-PAGE of V21H1-DOS47 and V21H4-DOS47. Bands labeled in red with 1, 2, or 3 are cluster numbers. Lane 1: molecular weight ladder. Lane 2: high purity urease (HPU). Lanes 3 and 4: V21H1-DOS47. Lanes 5 and 6: V21H4-DOS47. **(B)** Size exclusion chromatograms of V21H1, V21H4, HPU, V21H1-DOS47, and V21H4-DOS47.

After V21H1 and V21H4 were cross-linked, they were then conjugated to urease to generate V21H1-DOS47 and V21H4-DOS47, respectively. In both cases, sulfhydryl chemistry was used to conjugate the antibody-linker to urease. SDS-PAGE was performed to evaluate both conjugates (Figure 5A). During conjugation, each of the six monomeric urease subunits could potentially be cross-linked with up to five antibody molecules; therefore, under denaturing SDS-PAGE conditions, both V21H1-DOS47 and V21H4-DOS47 would be expected to generate a pattern of six discrete bands ranging from ~90 to 180 kDa. However, it appears that a maximum of four antibodies are conjugated per urease, as only five discrete bands are observed (Figure 5A, cluster 1). This suggests that one of the five cysteine residues on the surface of urease has little or no ability to react with maleimide. In addition to the expected five discrete bands, additional clusters of bands are observed for both V21H1-DOS47 and V21H4-DOS47. For V21H1-DOS47, two additional clusters are apparent. Cluster 2 (effective MW from ~200 to 250 kDa) and cluster 3 (effective MW >300 kDa) are likely urease dimers and polymers generated by V21H1 species carrying multiple SM(PEG)_2_ cross-linkers. While these higher molecular weight species could be composed of multiple native urease molecules, the low levels (less than 5%) of dimer and polymer peaks observed by SEC (Figure 5B) suggests that the majority of these species are composed of inter-subunit linkages of a single native urease molecule and not inter-molecular linkages. For V21H4-DOS47, since only the C-terminal cysteine is activated by BM(PEG)_2_, theoretically only one band cluster should be present. However, as demonstrated in lanes 5 and 6, an additional cluster is observed in the V21H4-DOS47 lanes (MW ≥ 150 kDa). The second cluster could be composed of non-covalent dimers that form as the conjugated subunits migrate in the gel. This was confirmed by SDS-PAGE capillary electrophoresis (not shown) in which no dimer clusters were observed. Therefore, V21H4-DOS47 does not contain cross-linked urease dimers or polymers.

SDS-PAGE was also used to determine the antibody:urease conjugation ratio (CR) for each native urease hexamer–antibody conjugate. Band intensities (Figure 5A) in cluster 1 depend upon the relative abundance of urease monomers linked to different numbers of antibody molecules. ImageLab software was used to generate histograms corresponding to band intensities and to integrate the peak areas of each histogram. The CR for native urease hexamers was calculated as follows:

$$\text{CR}=6\times \left({\text{PK}}_{1}\times 0+{\text{PK}}_{2}\times 1+{\text{PK}}_{3}\times 2+{\text{PK}}_{4}\times 3+{\text{PK}}_{5}\times 4\right)/\left({\text{PK}}_{1}+{\text{PK}}_{2}+{\text{PK}}_{3}+{\text{PK}}_{4}+{\text{PK}}_{5}\right)$$

where PK*_i_* (*i* = 1–5) is the peak area of the urease monomer linked with *i* − 1 antibody molecules.

Although there is a variable number of antibodies conjugated to each urease monomer, one would predict less variability in the number of antibodies per urease hexamer, as the monomers randomly cluster to form hexamers. This was confirmed by SEC of native V21H4-DOS47 in which the conjugate is observed to migrate as a tight peak (Figure 5B). The V21H4-DOS47 conjugation method reproducibly produced conjugates with 8.7–9.2 antibodies per urease (based on three batches).

The purities and the effective molecular weights of the antibodies, HP urease, and conjugates were assessed by SEC under native conditions (Figure 5B). As expected, V21H1 and V21H4 antibodies elute at comparable times (35.9 min). Free HP urease elutes at 26 min. As antibody molecules are linked to urease molecules for both V21H1-DOS47 and V21H4-DOS47, making the conjugates larger than free urease, the conjugates elute earlier than free urease. However, it is interesting that V21H1-DOS47 elutes 1 min before V21H4-DOS47 (22.80 vs 23.80 min). Both conjugates have nearly identical CRs (8.8 antibodies/urease for V21H1-DOS47 and 8.7 antibodies/urease for V21H4-DOS47). The V21H4 antibody has three more amino acids (159.20 Da) than V21H1; however, the theoretically larger V21H4-DOS47 conjugate appears smaller in effective molecular size in SEC than its counterpart V21H1-DOS47. This implies that V21H4-DOS47 is more compact than V21H1-DOS47 under native conditions. The majority of each species is in the monomeric form, with small dimer peaks appearing in front of each monomeric peak. It is notable that the V21H1-DOS47 conjugation procedure requires a SEC step in order to achieve high purity (96%). The SEC step removes urease polymers that are generated by V21H1 antibodies activated by two cross-linkers. However, the SEC step is not necessary to produce V21H4-DOS47, as V21H4 antibodies are activated by one cross-linker only. For V21H4-DOS47, a purity of greater than 97% is typically achieved using only diafiltration to remove unbound V21H4 antibody. As SEC methods are not easily transferred to large-scale GMP processes, it would be technically more difficult and expensive to produce V21H1-DOS47 for clinical use.

### Activity of V21H1-DOS47 and V21H4-DOS47

**Figure 6:**
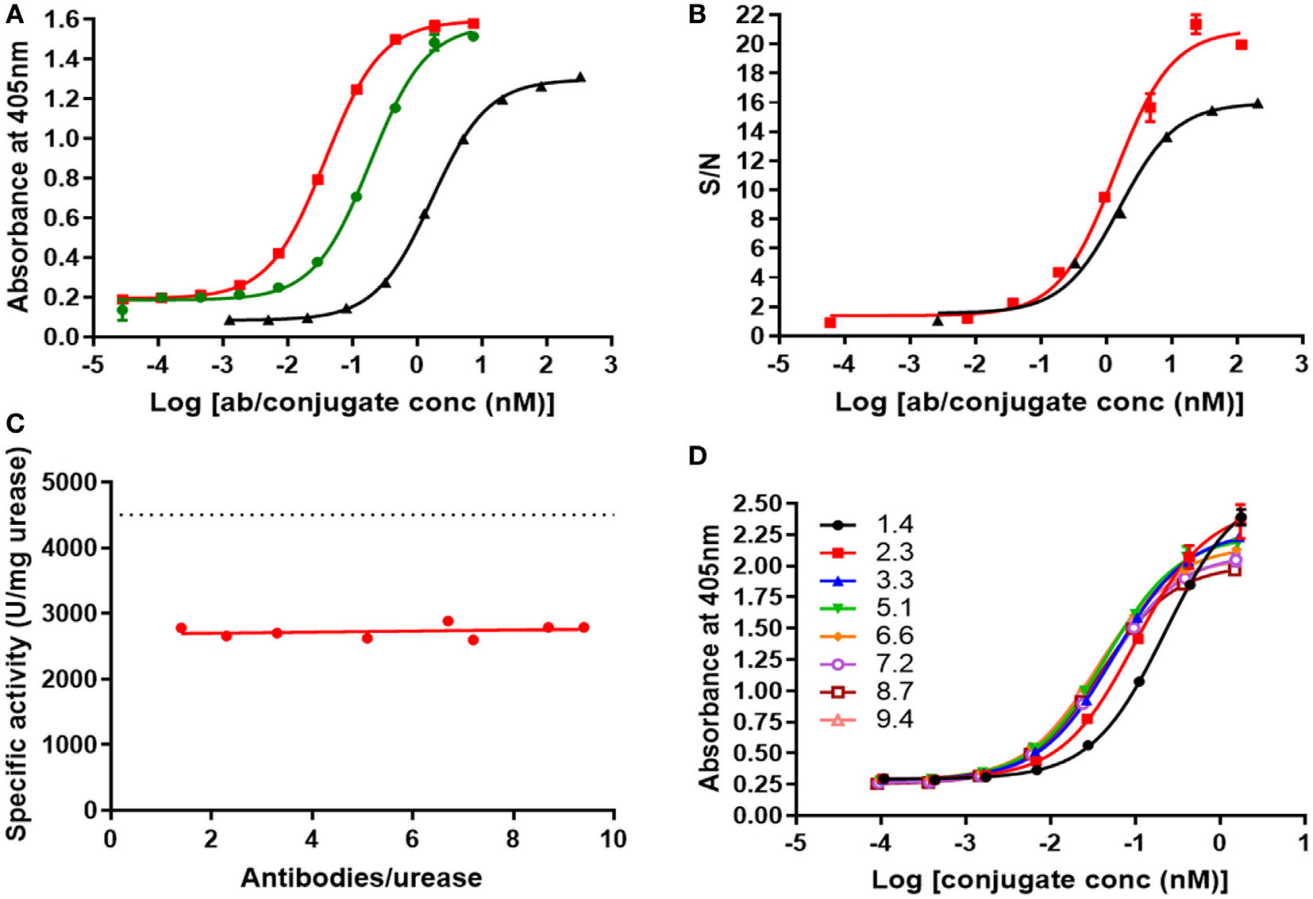
**(A)** ELISA of biotin-V21H4 (black), V21H1-DOS47 (green), and V21H4-DOS47 (red) binding to recombinant VEGFR2. Results shown are representative of two to five experiments performed for each sample and are presented as the means and SE of samples tested in triplicate. **(B)** Binding of biotin-V21H4 (black) and V21H4-DOS47 (red) to VEGFR2 expressed by 293/KDR cells. Binding was quantified by flow cytometry. Results shown are representative of two to three experiments performed for each sample and are presented as the means and SE of samples tested in duplicate. **(C)** Urease enzyme activity of V21H4-DOS47 at different antibody/urease conjugation ratios (CRs). The dotted line represents unconjugated urease activity. **(D)** ELISA of V21H4-DOS47 with different antibody–urease CRs binding to recombinant VEGFR2/Fc. Results shown are representative of two experiments performed for each sample and are presented as the means and SE of samples tested in duplicate.

An ELISA assay was performed to evaluate the binding of V21H1-DOS47 (9.2 antibodies/urease), V21H4-DOS47 (8.8 antibodies/urease), and biotin-V21H4 to recombinant VEGFR2/Fc (Figure 6A). V21H4-DOS47 (EC_50_ = 44 pM) binds to VEGFR2/Fc with approximately five-fold higher affinity than does V21H1-DOS47 (EC_50_ = 226 pM). As a substantial amount of V21H1 was conjugated to urease *via* the lysine present in CDR2, this is not surprising. V21H4-DOS47 also binds to VEGFR2/Fc with approximately 40-fold higher affinity than does V21H4 antibody alone (EC_50_ = 1.8 nM). This is most likely due to the multivalent nature of the conjugate. As V21H4-DOS47 is the superior conjugate, subsequent characterization was performed for V21H4-DOS47 only.

The ability of V21H4 antibody and V21H4-DOS47 conjugate to bind to cells expressing VEGFR2 (293/KDR) was evaluated by flow cytometry (Figure 6B). Biotin-V21H4 (EC_50_ = 1.6 nM) binds to 293/KDR cells with a similar affinity as to recombinant VEGFR2/Fc (EC_50_ = 1.8 nM, Figure 6A). This suggests that the VEGFR2 antibody epitope is equally accessible in recombinant VEGFR2/Fc in the ELISA assay and on the cell surface of 293/KDR cells. Interestingly, the binding of V21H4-DOS47 (EC_50_ = 1.2 nM) to the 293/KDR cells is very similar to the binding of biotin-V21H4 antibody to these cells (EC_50_ = 1.6 nM). Although there was an improved affinity observed for V21H4-DOS47 compared to V21H4 antibody in the ELISA assay with VEGFR2/Fc, this was not observed for cell binding. This suggests that the density of VEGFR2 expressed on the surface of 293/KDR cells is lower than in the wells of the ELISA plate.

Several factors contribute to determination of an ideal antibody/urease CR. During the conjugation reaction, the urease molecule is altered by linkage to the V21 antibody; therefore, depending on the CR, urease enzyme activity could be affected. On the other hand, the avidity of the antibody–urease complex increases as more antibodies are coupled to urease. To evaluate the effects of CR on both the urease enzyme activity and on binding activity, V21H4-DOS47 conjugates with different CRs (1.4–9.4 V21H4 per urease) were produced by adjusting the V21H4/HPU molar ratios.

The activity of unmodified urease is approximately 4,500 U/mg. When antibody is conjugated to urease, approximately 40% of the activity is lost (Figure 6C). However, the urease enzyme activity is independent of the number of antibodies conjugated, as activity remains consistent at all CRs tested. An ELISA assay using recombinant VEGFR2/Fc was performed to evaluate the binding of conjugates with different numbers of antibodies per urease (Figure 6D). When increasing from 1.4 to 2.3 antibodies per urease, the binding of the conjugate to VEGFR2/Fc improves, as indicated by a decrease in EC_50_ values from 226 to 93 pM. Addition of one more antibody (3.3 antibodies/urease) further reduces the EC_50_ to 58 pM. However, addition of subsequent antibodies/urease has a limited benefit: with 9.4 antibodies per urease, the EC_50_ is 31 pM. Thus, there is only a slight improvement in affinity when greater than 3.3 antibodies per urease are present. Thus, a CR of 3.3 antibodies per urease is sufficient for optimal urease activity and conjugate binding.

### Additional Characterization of V21H4-DOS47

**Figure 7:**
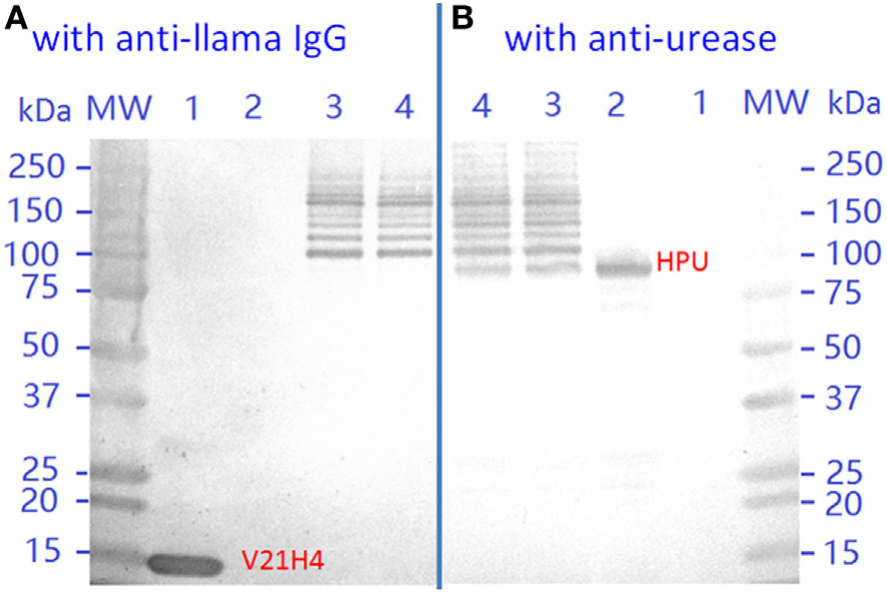
Western blot of V21H4, high purity urease (HPU), and V21H4-DOS47. Blots were probed with **(A)** an anti-llama antibody or **(B)** an anti-urease antibody. Lane MW: molecular weight ladder. Lane 1: V21H4. Lane 2: HPU. Lanes 3 and 4: V21H4-DOS47.

Dual-panel Western blotting (Figure 7) of V21H4-DOS47 was performed to confirm the banding pattern seen by SDS-PAGE. In Western blotting, the dimer and polymer clusters formed in-gel are more prominent than they appeared in SDS-PAGE (Figure 5A). When probed with anti-urease antibody, the urease band is visualized at molecular weight ~85 kDa and the bands of urease subunits bound to 1–4 antibodies match with the pattern seen by SDS-PAGE. When probed with an anti-llama antibody, the free urease subunit band is not observed and the antibody–urease conjugate bands are seen in the same pattern as when probed with an anti-urease antibody. The ability of V21H4-DOS47 to be visualized by both the anti-llama and anti-urease antibodies demonstrates the presence of both species in the conjugate.

**Figure 8:**
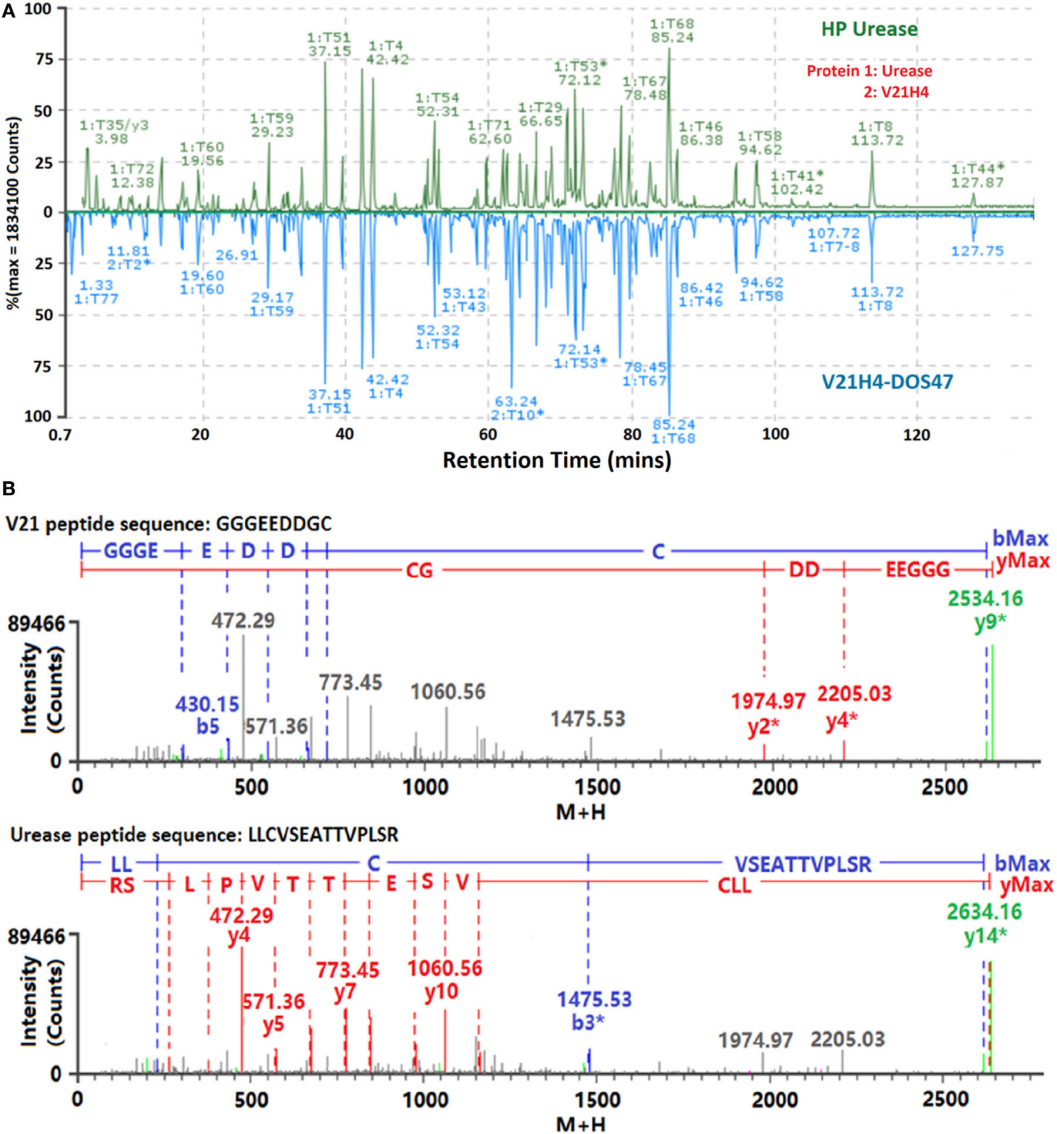
**(A)** Screen snapshots of raw LC-MS (TIC) chromatograms of tryptic digests of HP urease (top) and V21H4-DOS47 (bottom) samples processed by BiopharmaLynx software. **(B)** Screen snapshots of *b*/*y* fragment profiles of conjugation site UC_824_–VC_136_ mapped as the V21H4 peptide GGGEEDDGC (top) modified by UC_824_-BM(PEG)_2_ and as the urease peptide LLCVSEATTVPLS (bottom) modified by VC_136_-BM(PEG)_2_.

ESI-LC-MS^E^ peptide mapping analysis was employed to confirm the identities of V21H4 and urease and to identify the conjugation sites of V21H4-DOS47. The LC-MS (TIC) chromatograms of V21H4-DOS47 and HPU are shown in Figure 8A. The identified peptides covered 100% of V21H4 and urease protein sequences with mass match errors less than 4 ppm. All identified peptides with greater than three residues were confirmed by elevated energy MS/MS with at least half of the *b*/*y* ions identified. Since only the C-terminal GGGEEDDGC (837.2446 Da) of V21H4 is linked to different cysteine-carrying peptides of urease, the conjugation sites (denoted as UC*_x_*-VC_136_, where *x* is the amino acid in the urease protein sequence) are those urease peptides modified by GGGEEDDGC-BM(PEG)_2_ (1,145.3453 Da). To identify those covalent conjugation sites, ESI LC-MS^E^ raw data of the tryptic digests from V21H4-DOS47 samples were processed by BiopharmaLynx and searched against the urease protein sequence with a variable modifier of 1,145.3453 Da applied to all 15 urease cysteine residues. In order to assess the relative frequency of each conjugation site, the peptide intensities of the conjugated peptides UC*_x_*-VC_136_ were compared with the sum intensities of all the peptides related to UC*_x_* to generate the percentage of conjugation (Table 3). Among the 15 cysteine residues of each urease subunit, only 4 were conjugated (consistent with bands observed by SDS-PAGE, Figure 5A). The most accessible cysteine is C_824_ (26.7%), followed in order by C_663_ (4.2%), C_59_ (2.6%), and C_207_ (0.6%). No conjugation was detected to cysteine residue C_592_, which is essential to urease enzyme activity. This is consistent with the observation that urease activity is comparable at all CRs (Figure 6B).

Conjugation sites were also identified as V21H4 peptides modified by -UC*_x_* (UC*_x_* + 308.1008 Da). This was done by searching the V21H4 antibody protein sequence against -UC*_x_* as the variable modifier to the C-terminal cysteine of V21H4 (Table 3). Among the identified tryptic peptides, 0.4% of them were unmodified (T:012). This trace amount of peptide could be the portion of V21H4 activated by the cross-linker through C_23_ and C_97_ of the core sequence. Alternately, this peptide could be a trace amount of V21H4 attached to the C-terminal half cystamine that was not de-protected in the TCEP reduction step. These results are consistent with those observed with urease peptides modified by -VC_136_. Most of the V21H4 C-terminal cysteine was conjugated to urease *via* C_824_ (59%), with less conjugation at C_663_ (27%), C_59_ (12%), and C_207_ (1.2%).

The identities of the conjugation sites were confirmed with *b*/*y* ion mapping of urease and V21H4 peptides. Among the 16 possible V21H4 *b*/*y* ions, only a few (4–7) were identified from the three major urease conjugation sites. This could be a result of the ESI ionization property of the GGGEEDDGC residues, which causes a lack of positive charge center in the ionization environment. However, the MS/MS *b*/*y* fragment profiles (Figure 8B) can be assessed by looking at both V21H4 and urease proteins. As an example, the conjugated peptide UC_663_-VC_133_ whose sequence is (LLCVSEATTVPLSR)-linkage-(GGGEEDDGC) and which has a peptide mass of 2,633.1472 was identified with a mass match error of 2.1 ppm by searching it as LLCVSEATTVPLSR, a urease peptide modified with (GGGEEDDGC)-linkage (1,145.3453 Da) from the V21H4 side as the modifier. The same peptide was also identified with a mass match error of 2.1 ppm by searching it as GGGEEDDGC, a V21H4 C-terminal peptide modified with (LLCVSEATTVPLSR)-linkage (1,795.9026 Da) from the urease side as the modifier. The MS^E^ collision induced MS/MS spectrum of this conjugated peptide was mapped with 13 *b*/*y* fragment ions from the urease side by searching it as a urease peptide modified with the modifier from the V21H4 side. The same spectrum was also mapped with 7 *b*/*y* ions from the V21H4 side by searching it as a V21H4 peptide with the modifier from the urease side.

### Specificity of V21H4-DOS47

**Figure 9:**
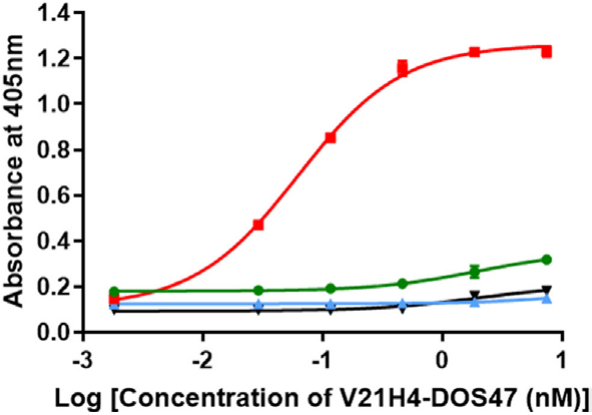
ELISA of V21H4-DOS47 binding to VEGFR1, VEGFR2, and VEGFR3. Green line is VEGFR1. Red line is VEGFR2. Blue line is VEGFR3. Black line is no VEGFR. Results shown are representative of two experiments performed for each sample and are presented as the means and SE of samples tested in triplicates.

An ELISA was performed to evaluate antibody specificity *via* binding of V21H4-DOS47 to VEGFR1, VEGFR2, and VEGFR3 (Figure 9). Recombinant Fc fusion proteins of VEGFR1, VEGFR2, and VEGFR3 were captured on 96-well plates coated with goat anti-human IgG/Fc. After incubations with various concentrations of V21H4-DOS47, binding was detected using a rabbit anti-urease antibody, goat anti-rabbit-AP, and substrate. As expected, V21H4-DOS47 binds well to VEGFR2; however, there is minimal, if any, binding to VEGFR1 or VEGFR3. The binding observed to VEGFR1 (shift upwards of the curve) is due to non-specific binding of one of the detection reagents to VEGFR1.

## Discussion

Antibody–drug conjugates are emerging as a promising class of anti-cancer drugs. By delivering drugs directly to the target site, non-specific side effects are reduced. We have previously described the production and characterization of L-DOS47, an ADC composed of the enzyme urease and an anti-CEACAM6 antibody (9). L-DOS47 is currently in phase I/II trials for the treatment of non-small cell lung cancer. In this study, we expand our knowledge of this class of drugs with the generation and characterization of V21H4-DOS47, which targets VEGFR2. Although L-DOS47 and V21H4-DOS47 were both generated by conjugating urease to a llama single domain antibody, considerable optimization was required to produce a successful V21H4-DOS47 conjugate. For example, initial V21-DOS47 conjugates generated using the same linker as in L-DOS47, SIAB, were unsuccessful due to poor binding of the conjugate (data not shown). SIAB is a short and rigid linker and it would seem that the orientation of V21 on the surface of the urease was sub-optimal with this linker. Upon switching to the PEG_2_ class of linkers, which are relatively long and flexible, binding activity of the conjugate was considerably improved.

In this study, we developed procedures to conjugate and purify the V21-DOS47 immunoconjugate that are suitable for large-scale cGMP production. Single domain camelid antibodies are ideal for use in generating antibody–enzyme conjugates. Their small molecular size allows them to be produced affordably in large amounts. Importantly, they can also be easily modified by adding a short amino acid tag at the C-terminus. The tag serves several purposes, including modification of the antibody pI, promotion of targeted antibody expression, and addition of a specific reaction site. Since the pI of urease is in the 4.8–5.1 range, an antibody–urease conjugate generated with the unmodified core antibody would produce a conjugate with a pI of approximately 7. At this pI, the conjugate is unstable and forms precipitates during and after conjugation. The addition of a short C-terminal peptide tag adjusts the pI of the antibody from 8.75 to 5.43 leading to a conjugate with a pI between 4.8 and 5.5 which is stable during conjugation and purification. The C-terminal tag also improves the yield of antibody production by targeting expression to bacterial inclusion bodies. This allowed antibody purification using only ion exchange chromatography. As the V21 sequence contains two lysine residues in the CDR2 and CDR3 sequences, respectively, lysine-to-sulfhydryl cross-linking chemistry could modify these lysine residues, compromising the binding affinity of the conjugate to its target antigen. For this reason, a C-terminal cysteine residue was included in the C-terminal tag of V21H4 for use in sulfhydryl-to-sulfhydryl cross-linking chemistry. LC-MS^E^ characterization confirmed the modification of the CDR2 lysine residue by lysine-to-sulfhydryl cross-linking chemistry and an ELISA binding assay confirmed that the affinity of the V21H4-DOS47 produced by sulfhydryl-to-sulfhydryl cross-linking chemistry was approximately five-fold stronger than that of the V21H1-DOS47 conjugate produced by lysine-to-sulfhydryl cross-linking chemistry.

Although the addition of a C-terminal cysteine residue proved extremely useful in the conjugation of V21H4-DOS47, this method cannot be generalized and used for all llama single domain antibodies. The sulfhydryl-to-sulfhydryl chemistry uniquely targets the C-terminal cysteine only because the core cysteine residues are joined in a disulfide bond, and thus unavailable for modification. When working with other llama antibodies, it will be necessary to evaluate the status of any core cysteine residues before determining if this strategy can be used.

Protein refolding can be a slow and unreproducible process. Typically, refolding is performed by dilution or dialysis and the process can take several days. In addition, yield is generally low (25). The introduction of a DTT/cystamine redox couple led to a short and reproducible refolding process that generated high yields of active V21H4 antibody, which is very important for large-scale production.

One benefit of conjugating antibodies to urease is the apparent increased affinity of the conjugate compared to antibody alone. By clustering multiple antibodies per urease, avidity increases as the relative off-rate of the complex is slower than for free antibody. However, the improvement in antibody avidity must be balanced by the potential detrimental effects of adding antibody to urease, including impairment of urease activity and increased immunogenicity of the conjugate. In addition, high CRs increase production costs and complexity. Each antibody–urease conjugate may have a different ideal CR, as the availability of the target antigen differs and the orientation and activity of the antibody presented on the urease surface changes with different conjugation chemistries. In this study, we observed little improvement in antigen binding at CRs greater than 3.3. This is in contrast to L-DOS47, in which binding increased until eight antibodies were conjugated per urease. The use of a more flexible linker to generate V21H4-DOS47 compared to L-DOS47 may partially explain this difference, as the antibodies may be more accessible to target antigen. However, the difference between the two conjugates is most likely due to the fact that AFAIKL2, the antibody component of L-DOS47, has a much lower affinity for its target antigen than does V21 for VEGFR2 (data not shown). Thus, antibody multimerization has a more pronounced effect for AFAIKL2 than for V21. Future experiments evaluating the cytotoxic activity of V21H4-DOS47 will provide further important information required to determine the optimal CR.

We have extended our knowledge of antibody–urease conjugates by generating two versions of the V21 antibody and conjugating these antibodies to urease using two different linker chemistries. V21H4-DOS47 can be generated in an efficient and controlled manner, using methods that are scalable and amenable to cGMP procedures. We are currently evaluating V21H4-DOS47 as a clinical candidate for cancers that express high levels of VEGFR2.

## Author Contributions

BT made a substantial contribution to the design of the work; performed, analyzed, and interpreted experiments, and drafted and critically revised the manuscript. WW made a substantial contribution to the design of the work, performed, analyzed and interpreted experiments, and critically revised the manuscript. MU made a substantial contribution to the analysis and interpretation of the work and critically revised the manuscript. PW and HC each made substantial contributions to the conception and design of the work and critically revised the manuscript.

## Conflict of Interest Statement

The authors declare the following competing financial interests; BT, WW, MU, and HC are employees of Helix BioPharma Corporation. PW is an employee of Helix ImmunoOncology Sp. z o.o.
